# Three new and one little-known species of Hypogastruridae (Collembola) from Russia’s northeast

**DOI:** 10.3897/zookeys.1005.54882

**Published:** 2020-12-18

**Authors:** Anatoly Babenko, Boris Efeykin, Mikhail Bizin

**Affiliations:** 1 Severtsov Institute of Ecology & Evolution, Russian Academy of Sciences, Leninski pr. 33, 119071, Moscow, Russia Severtsov Institute of Ecology & Evolution, Russian Academy of Sciences Moscow Russia; 2 Kharkevich Institute for Information Transmission Problems, Russian Academy of Sciences, Bol’shoi Karetnyi per. 19, 127051, Moscow, Russia Kharkevich Institute for Information Transmission Problems, Russian Academy of Sciences Moscow Russia

**Keywords:** α-taxonomy, coastal communities, DNA sequencing, East Palaearctic, *
Hypogastrura
*, springtails, *
Xenylla
*

## Abstract

Three new species, *Hypogastrura variata***sp. nov.**, *Xenylla aculeata***sp. nov.**, and *X. arnei***sp. nov.**, are described based on material from coastal communities of the Sea of Okhotsk, northeast Russia. Taxonomic remarks concerning a little-known species, *H. yosii* Stach, 1964, found in coastal wrack sediments on Kunashir Island, Kuriles are also given. COI sequences of the above species are analysed, thus allowing for their species statuses to be confirmed.

## Introduction

The first information concerning the collembolan faunas of the Far Eastern regions of Russia was obtained during the expedition of N.A.E. Nordenskjöld aboard the ’Vega’ along the northern coasts of Eurasia to the Commander Islands (1878–1879). The material collected by that expedition on the northern coast of Chukotka was published by Schött (1893), who recorded slightly fewer than 20 species of Collembola from the region. Currently, this number totals 466 species (Kuprin and Potapov 2018) and continues to increase. This paper is devoted to descriptions of three new species of the family Hypogastruridae from the coastal communities in the northern part of the Sea of Okhotsk. Moreover, their statuses are confirmed using both morphological and molecular studies.

### Abbreviations

**Abd.1–6** abdominal segments;

**A1, A7** tenent seta in distal whorl of setae on tibiotarsi;

**a-, m-, p-setae** setae of anterior, medial, and posterior rows on terga, respectively;

**A–E papillae** and labial papillae and associated guards on the labial palp, according

**a-, b-, d-, e-guards** to Fjellberg (1999);

**Ant.1–4** antennal segments;

**L.1–6** maxillary lamellae;

**l.p.** lateral process on the labial palp;

**MSPU**Zoology and Ecology Department of the Moscow State Pedagogical University;

**ms** microsensillum/-a;

**or** organite on antennal tip;

**PAO** postantennal organ;

**S3**, **S7–S9** antennal sensilla;

**VT** ventral tube;

**U_3_** unguis of leg 3.

## Methods of molecular analysis

DNA was isolated from specimens fixed in 96% ethanol using Holterman’s technique (Holterman et al. 2006), with the addition of proteinase K and mercaptoethanol in the lysing solution. Sequences of the cytochrome oxidase subunit I (COI) gene were amplified using an EncycloPlus PCR Kit (Evrogen, Russia) with primers from the Table 1. The standard PCR reaction protocol of the Canadian Center for DNA Barcoding was used for amplifications (http://www.dnabarcodes2011.org/conference/preconference/CCDB-Amplification-animals.pdf). Polymerase chain reaction (PCR) products were visualised in gel, cut out, and cleaned using the SV Gel and PCR CleanUp System kit (Evrogen, Russia). DNA sequencing was performed at the Genome Centre for Collective Use in the Severtsov Institute of Ecology and Evolution of Russian Academy of Science (Moscow, Russia). The sequences were combined and aligned using ClustalX software after the addition of sequences from the GenBank. Distance analyses were performed with MEGA6 (Tamura et al. 2013) with the Kimura-2 parameter model (Kimura 1980) to estimate genetic distances. All sequences were deposited into the GenBank.

**Table 1.** Species used for molecular study, primers, and GenBank accession numbers of the sequences.

Species	Forward primer	Reverse primer	COI sequence number	Sequence size
*Hypogastrura variata* sp. nov.	colfol-for: tttcaacaaatcataargayatygg	colfol-rev: taaacttcnggrtgnccaaaaaatca	KY066780 KY066781 KY066782 KY066783	660 bp
*Hypogastrura yosii* Stach, 1964	colfol-for: tttcaacaaatcataargayatygg	colfol-rev: taaacttcnggrtgnccaaaaaatca	KY066784 KY066785 KY066786	660 bp
*Xenylla arnei* sp. nov.	LCO1490_t1: tgtaaaacgacggccagtgg tcaacaaatcataaagatattgg	HCO2198_t1: caggaaacagctatgactaaacttc agggtgaccaaaaaatca	KY066787 KY066788 KY066789	652 bp

## Species descriptions

### Hypogastrura variata sp. nov.

Taxon classificationAnimaliaPoduromorphaHypogastruridae

5DF0D55C-6328-5E05-B8A7-4245E04EE31B

http://zoobank.org/28CCD965-FD16-4F17-9817-C1FA1A773A51

Figs 1–19 , 20–23

#### Type material.

***Holotype*** Russia, North-East • ♂; Magadan Province, Ola; 59°36.19'N, 151°21.72'E; maritime marsh with *Carex subspataceae*; July 2017; M. Bizin and B. Efeykin leg.

***Paratypes*** Russia, North-East • ♀; same data as for holotype • 13 ♀♀, 6 ♂♂ and 10 juveniles; same region, but Tauisk; 59°44.07'N, 149°23.32'E; maritime marsh with *Puccinellia phryganodes*; July 2017; M. Bizin and B. Efeykin leg. The types are deposited in MSPU.

#### Additional material.

more than 300 specimens (alcohol), mainly from the *P. phryganodes* plant association of the same region. Several specimens from this material were sequenced (Table 1). Their partial COI genes were amplified and deposited in the GenBank under the sample ID: KY066780–KY066783.

#### Diagnosis.

A species of the genus *Hypogastrura* Bourlet, 1839, with four weakly differentiated, curved, sensory setae (one dorsal and three external) on Ant.4, the relatively clearly differentiated dorsal setae, the tridentate retinaculum, the basal lamella on the unguiculus, one tenent seta on all tibiotarsi, the partly reduced furca with four or five posterior setae, and the highly variable shape of the mucro.

#### Description.

**Figures 1–19.**
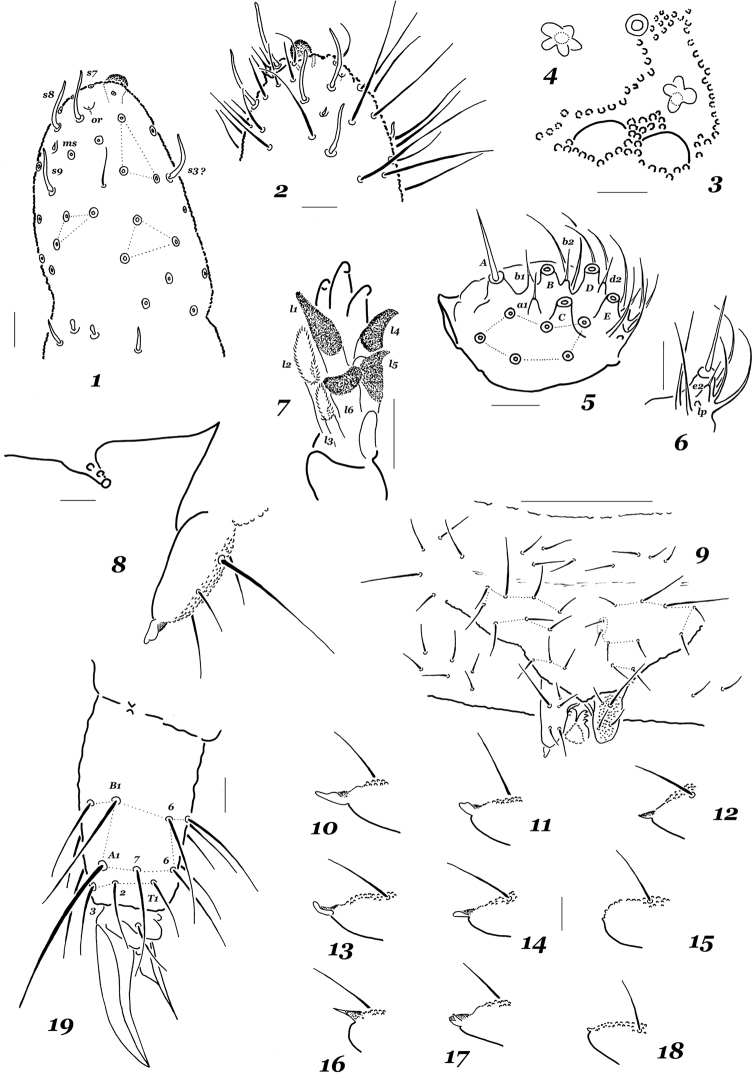
*Hypogastrura variata* sp. nov. **1** sensorial equipment of Ant.3 and Ant.4 **2** tip of Ant.4 **3**PAO and nearest ocelli **4**PAO, different specimen **5** labial palp **6** labial papilla E **7** maxillary head **8** furca and retinaculum, lateral view **9** sternum of Abd.4 **10–18** mucro, different specimens **19** tip of leg 3. Scale bars: 0.1 mm (**9**), 0.01 mm (**1–8, 10–19**).

Length of males 1.2–1.5 mm, females 1.2–1.8 mm, holotype 1.41 mm long. Colour dark, bluish black, not paler ventrally. Granulations fine and uniform, with 14–18 granules between setae p1 on Abd.5. Ant.4 with a simple apical bulb and four weakly differentiated, curved, sensory setae (one dorsal [S3 ?] and three external [S7, S8, and S9), subapical organite (or) and microsensillum (ms) present as usual (Figs 1, 2). Ant.3 organ typical of the genus, with all usual sensorial elements: two outer guards, two inner sensilla and a lateral microsensillum. Ant.1 and Ant.2 with seven and 13 setae, respectively. Head with 8+8 virtually equal ocelli. PAO slightly smaller than nearest ocelli, usually with four subequal lobes (range 3–6), an accessory boss not developed (Figs 3, 4). Distal edge of labrum with six low papillae, setal formula of labrum, 4/554. Labium typical of the genus, with all common papillae (A–E), 14 guards (a1, b1, b2, d2, and e2 shorter and set on low papillae) and six proximal setae, lateral process (lp) rudimentary (Figs 5, 6). Basomedial field of labium (submentum) with four setae, basolateral field (mentum) with five setae, as usual. Head with 3+3 postlabial setae present along ventral line. Maxillary head unmodified, of general generic type, L.1 hardly longer than maxillary teeth, L.2 and L.3 with short marginal filaments and usually few denticles, all other lamellae densely covered with fine denticles (Fig. 7), outer lobe simple, with two sublobal hairs.

**Figures 20–23.**
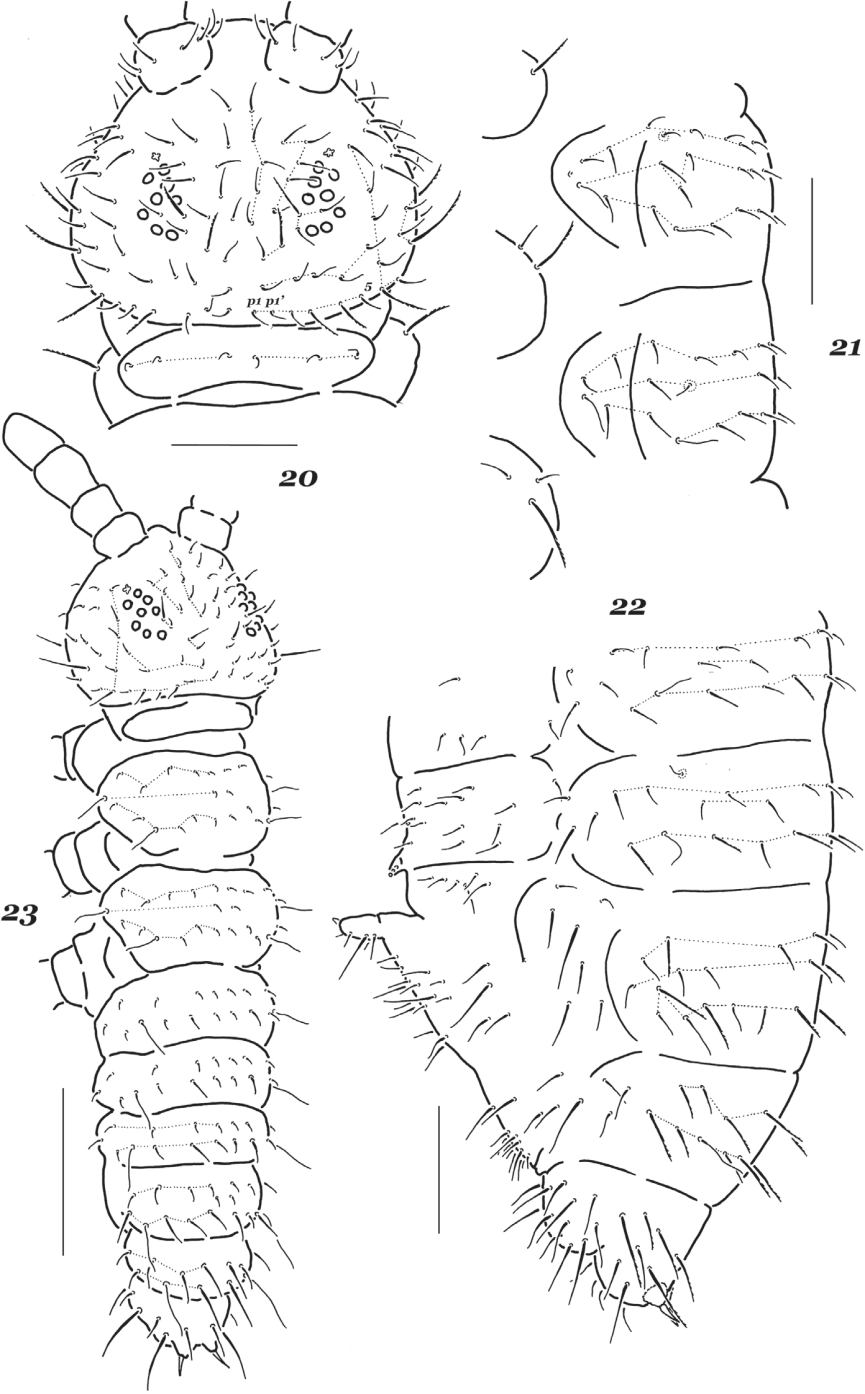
*Hypogastrura variata* sp. nov. **20** chaetotaxy of head and Th.1 **21** chaetotaxy of Th.2 and Th.3, lateral view **22** chaetotaxy of Abd.2–6, lateral view **23** chaetotaxy of juvenile, I instar. Scale bars: 0.1 mm.

*Dorsal chaetotaxy* typical of the genus (Figs 20–23). Most dorsal setae stout and finely serrate, those on abdominal tip (Abd.5 and Abd.6) clearly longer and rougher, sensorial setae thin and not especially long compared to ordinary ones. Main characteristics: detectible differentiation into micro- and meso- or macrosetae on all terga including head in adults (Figs 20–22) and, especially, juveniles (Fig. 23), usual presence of an additional seta in p-row on head (six p-setae totally) and abnormal variability (unusual shapes, absence or doubling of certain setae). Chaetotaxy of legs 1–3 as follows: upper subcoxae – 1, 2, 3 (among them, one macroseta on each subcoxa); lower subcoxae – 0, 3, 3; coxae – (2)3, 3, 3; trochanters – 7(8), 7(8), 7; femora – 13–14, 13–14, 12–14; tibiotarsi – 19, 19, 18 setae, respectively. Tenent tibiotarsal setae (A1) of moderate length, ~ as long as 1.1–1.5 inner unguis edge, truncate or indistinctly clavate. Unguis slender and usually toothless, rarely an indistinct tooth present in midsection of inner edge. Unguiculus with a clear basal lamella, its apical filament reaching the middle of inner unguis edge or slightly above (Fig. 19). Ventral tube with 4+4 distal setae. Retinaculum with 3+3 teeth. Furca short (Figs 8, 9), mucro rudimentary, sometimes completely absent (often asymmetrically), its shape highly variable (Figs 10–18). Manubrial field with 10–12+10–12 ventral setae including one or two basolateral macrosetae (Fig. 9). Dens usually with four or five posterior setae (whole range 2–6), one of which ~ as long as dens+mucro or even longer (ratio = 0.9–1.2:1). Mucrodens slightly longer than inner edge of hind unguis (1.0–1.3 ×). Anal spines rather strong and slightly curved, set on high contiguous papillae.

#### Variability.

One of the most characteristic features of the new species is its high-level variability of such important morphological traits as the number of PAO lobes and dental setae, as well as the shape and presence of a mucro (Table 2). This may be assumed as a direct consequence of rather severe conditions of boreal maritime marshes.

**Table 2.** Variation of some important morphological characters in *Hypogastrura variata* sp. nov.

Number of dental setae	%		presence of a mucro	%		PAO lobes	%	
	adults	juveniles		adults	juveniles		adults	juveniles
3+3	–	20	0+0	10	20	4+3	12	–
3+4	3	40	1+0	47	30	4+4	28	70
4+4	30	40	1+1	43	50	4+5	56	30
4+5	37	–				5+6	4	–
5+5	27	–						
5+6	3	–						
Number of specimens studied	30	10		30	10		25	10

#### Etymology.

The name of the new species is intended to reflect the morphological variability.

#### Affinities.

Apart from the new species, there are only four congeners known in the world fauna that are characterised by a shortened furca with five or fewer dental setae, coupled with a tridentate retinaculum and only one tenent seta on each leg: *H. oreophila* Butschek, 1948, *H. exigua* Gisin, 1958, *H. mongolica* (Nosek, 1976), and *H. magistri* Babenko, 1994. The first two species are from high-montane habitats in the European Alps, and both have been recently redescribed (Skarżyński 2011). They are much smaller than *H. variata* sp. nov. (0.8 mm vs. 1.2–1.8 mm) and have short, undifferentiated, dorsal setae and a longer furca (dens+mucro/U_3_ with ratio ~ 2 vs. 1.0–1.3 in *H. variata* sp. nov.). In addition, *H. oreophila* is characterised by the presence of m-setae on Abd.5, a broadened maxillary L.1, an inner tooth on the unguis and a hook-like mucro with a broad outer lamella, whereas *H. exigua* shows a larger PAO (~1.5 ocellus) and more numerous setae on VT (5+5 vs. 4+4 in *H. variata* sp. nov.).

The two other similar species, *H. mongolica* and *H. magistri*, are known from mountainous regions of Central Asia (northern Mongolia and western Tuva). Of these, *H. magistri* can easily be distinguished due to the presence of six or seven curved sensilla on Ant.4 (vs. four in *H. variata* sp. nov.) and the presence of additional setae on Abd.4 and Abd.5. As regards *H. mongolica*, its comparison with *H. variata* sp. nov. is impossible, because the holotype, the only known specimen, of *H. mongolica* was immature (Skarżyński 2011). According to the original description (Nosek 1976) and redescription of the type (Skarżyński 2011), *H. mongolica* differs from *H. variata* sp. nov. in being smaller (0.6 mm long) and lighter in colouration, the “body clothed sparsely with short setae”, long tergal sensilla and an inner tooth on the unguis, but at least some of these characters may reflect its immature status. Nevertheless, *H. mongolica* and *H. variata* sp. nov. are unlikely to be synonymous from an ecological point of view alone, because their habitat preferences are drastically different: litter of a mountain forest vs. a saline maritime marsh.

There are another four known Palaearctic congeners which may be related to the above group: *H. capitata* Cassagnau & Delamare, 1955 (Lebanon), *H. verruculata* Rusek, 1967 (China), *H. ramia* Lee & Choe, 1979 (South Korea), and *H. pizzoci* Fanciulli & Dallai, 2008 (Italy). All of them are also characterised by the presence of a single tenent seta on each leg, and the unguiculus with a basal lamella and a tridentate retinaculum, but they all share a complete, less strongly reduced furca with six posterior setae.

#### Molecular data.

Unfortunately, the GenBank does not contain sequences of any of the above-mentioned species. Therefore, the isolated position of *H. variata* sp. nov. among fifteen taxonomic units of *Hypogastrura* present in the GenBank is not particularly surprising and may well serve as an additional confirmation of its independent status. Molecular data have shown that the divergences between all units considered are rather high (Table 3). The average interspecific divergence between all species is 26.3% (ranging from 15.9 to 36.4%), while it is 26.7% for *H. variata* sp. nov. and the other fourteen species (ranging from 23.0 to 31.4%). Nevertheless, it seems noteworthy that the molecular trees obtained fail to fully reflect the relationships within the genus *Hypogastrura* based on morphological evidence alone. The most apparent assumption explaining this fact is that the molecular data are still too scant to realistically construct reliable trees that would adequately reflect the real phylogenetic relationships within the genus.

**Table 3.** K2P distances in *Hypogastrura* species from GenBank and our sequences, measured in %.

	Species	Region	1	2	3	4	5	6	7	8	9	10	11	12	13	14
1	*H. arctandria*	Ontario														
2	*H. assimilis*	Ontario	20.2													
3	*H. yosii*	Kunashir	22.2	25.2												
4	*H. concolor*	Ontario	25.6	26.3	23.8											
5	*H. distincta*	Ontario	19.2	26.1	24.2	22.5										
6	*H. helena*	Alaska	25.7	28.2	28.5	23.5	21.5									
7	*H. macrotuberculata*	Ontario	22.7	29.9	29.4	29.3	21.1	28.6								
8	*H. socialis*	Estonia	23.0	26.8	26.9	29.0	24.3	24.3	27.7							
9	*H. reticulata*	Japan	30.3	34.8	34.2	32.2	26.3	28.4	28.4	31.4						
10	*H. sensilis*	Ontario	23.6	28.0	25.6	18.9	23.7	23.0	28.8	26.9	35.4					
11	*H. variata* sp. nov.	Magadan	23.0	26.8	26.9	29.0	24.3	24.3	27.7	25.2	31.4	26.9				
12	*H. subboldorii*	France	21.4	28.3	23.3	23.6	24.0	24.9	24.4	26.2	34.2	22.6	26.2			
13	*H. tooliki*	Alberta	26.6	28.3	27.6	25.0	28.3	26.0	28.3	27.2	28.3	25.4	27.2	26.1		
14	*H. vernalis*	France	27.0	24.6	25.3	26.2	26.5	27.7	23.8	28.7	36.6	23.9	28.7	27.6	27.6	
15	*H. viatica*	Churchill	24.2	26.2	24.8	18.1	23.4	25.3	31.4	26.6	36.4	15.9	26.6	22.7	30.0	26.0

#### Distribution and ecology.

*Hypogastrura variata* sp. nov. was collected in two neighbouring sites located on the northern shore of the Sea of Okhotsk, both in the vicinity of Magadan. It seems to inhabit a narrow belt of mudflat maritime marshes, i.e., a monodominant plant association *Puccinellietum phryganodis*, where it achieves very high abundance levels and is the most common collembolan species. Its occurrence in all other types of marsh in the study area was sporadic.

### Hypogastrura yosii

Taxon classificationAnimaliaPoduromorphaHypogastrura

Stach, 1964

91DF61B9-9A32-5F05-874C-CC8FB4C537CA

Figs 24–29

Syn.: Hypogastrura sheyangensis Jiang, Tang & Chen, 2007.

#### Material.

Russia • 3 ♂♂, 9 ♀♀ (slides) and ~ 50 specimens (alcohol); Kuril Islands, Kunashir; 43°42.91'N, 145°33.20'E; wrack beds; August 2017; K. Makarov leg. Several specimens from this material were sequenced (Table 1). Their partial COI genes were amplified and deposited in the GenBank under the sample ID: KY066784–KY066786.

#### Taxonomic remarks.

Stach’s (1964) original description of *H. yosii* was based on two specimens collected in forest sites in eastern China. Its real position within the genus had remained unclear until it was recently redescribed from the types (Jia et al. 2011) and synonymised with *H. sheyangensis* Jiang, Tang & Chen, 2007. The latter species was known from the coastal wetlands of the same region. Specimens from Kunashir Island fit rather well with the existing descriptions, but are significantly smaller, 0.7–0.9 mm vs. “up to 1.5 mm” in Chinese specimens.

**Figures 24–29.**
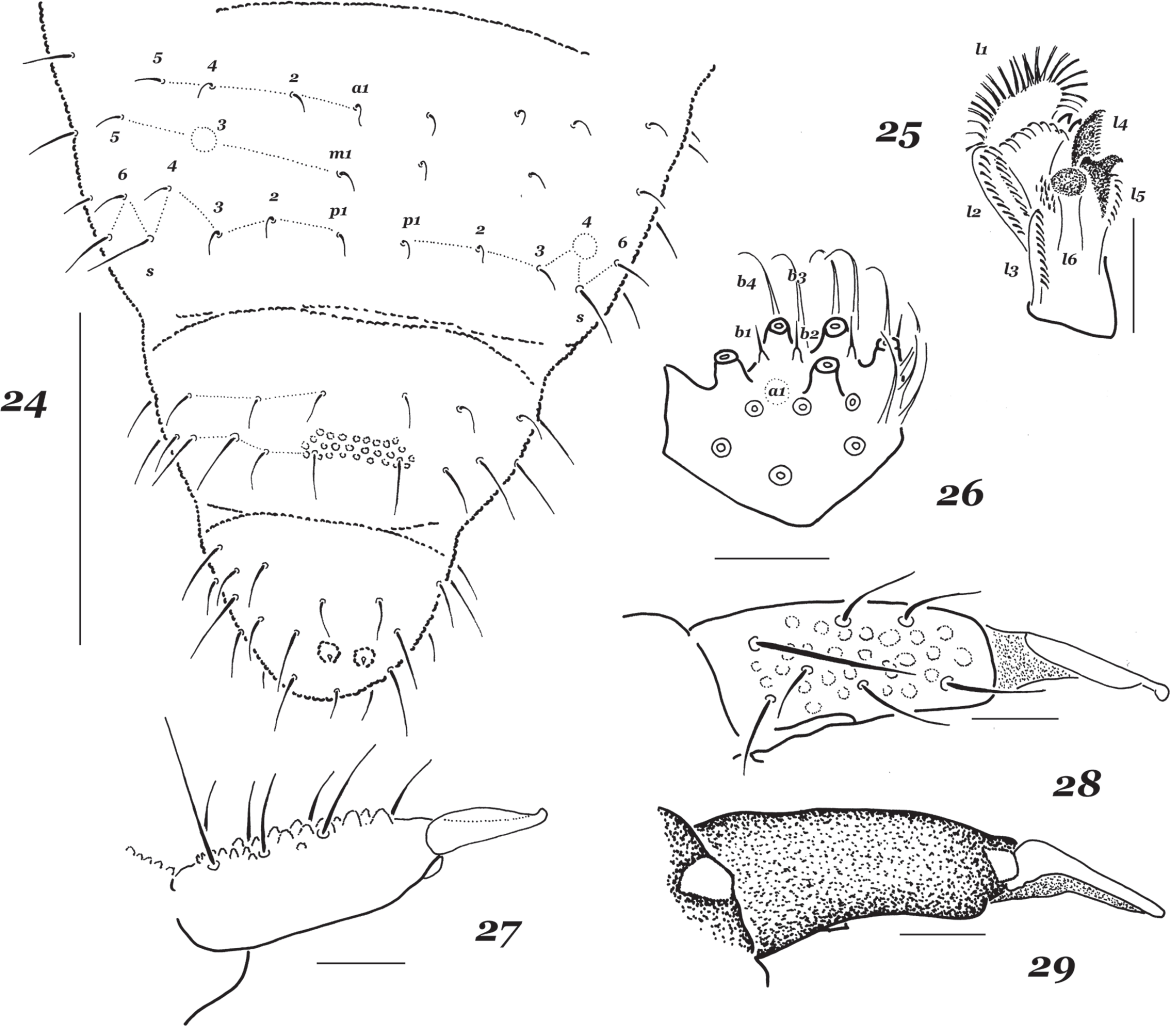
*Hypogastrura yosii* Stach, 1964 **24** dorsal chaetotaxy of Abd.4–6 **25** maxillary head **26** labial palp **27** dens and mucro, lateral view **28** dens and mucro, dorsal view **29** dens and mucro, ventral view. Scale bars: 0.1 mm (**24**), 0.01 mm (**25–29**).

Both recent descriptions (Jiang et al. 2007; Jia et al. 2011) considered *H. yosii* as being most similar to two Nearctic species, viz. *H. matura* (Folsom, 1916) and *H. utahensis* (Wray, 1953) (now a junior synonym of *H. promatro* (Wray, 1950) [see Bernard 2015]). This opinion was mainly based on the absence of seta p4 on Abd.4. In fact, this seta, set anteriorly to the p-row (almost aligned with m-setae), may occasionally be absent on one or both sides in many species of the *manubrialis*-group. This is also rather frequent in specimens of *H. yosii* from Kunashir Island (Fig. 24). In our view, *H. yosii* is closely related to the widespread *H. manubrialis* (Tullberg, 1869). Apart from the absence of a seta m2 on Th.2 (one of the most notable peculiarities of *H. manubrialis*), the similar number of sensilla on Ant.4, the relatively large PAO with secondary projections at the base of the lobes, and the short anal spines, both species are characterised by an almost identical structure of the maxillary head (except for lam.6, which in *H. manubrialis* has no denticles in the central part) (Fig. 25) and the absence of a a1 guard on the labial palp (Fig. 26). This short guard is present in most congeners studied, but not in the *manubrialis* group, in which it is present in only some species: *H. arctandria* Fjellberg, 1988, *H. assimilis* (Krausbauer, 1898), *H. vernalis* (Carl, 1901), and *H. promatro*, but it is absent from others: *H. manubrialis*, *H. yosii*, *H. serrata* (Ågren, 1904), and *H. rangkuli* Martynova in Martynova and Chelnokov 1975. Meaningful differences between *H. yosii* and *H. manubrialis* appear to be limited by maxillary lam.6 (see above), the presence of seta p’ on Ant.1 in *H. yosii* (a variable character in specimens from Kunashir, where only six of ten specimens examined show p’ seta on at least one antenna) and a characteristic mucro shape, i.e., a long and slender mucro without clear lateral lamellae in *H. manubrialis* or a mucro with an “upturned apex and [clear] outer lamella” in *H. yosii* (Figs 27–29).

### Xenylla aculeata sp. nov.

Taxon classificationAnimaliaPoduromorphaHypogastruridae

96402357-052B-5300-8079-46A314211762

http://zoobank.org/AC26673B-7C17-4B16-B800-9700FC9906BF

Figs 30–35

#### Type material.

***Holotype*** Russia, North-East • ♂; Magadan Province, “Magadan–Ola Road (closer to Ola) [~ 59°35.25'N, 151°08.00'E]; treeless bog with *Ledum*, *Arctostaphylos*, *Empetrum*, and *Betula* vegetation (sample taken in *Carex* and moss), 20.07.1979”; V. Behan leg. The type is deposited in MSPU.

#### Diagnosis.

A species of chaetotaxic group VI (b) of the genus *Xenylla* Tullberg, 1869, characterised by the presence of one sublobal seta on the maxillary outer lobe and a complete reduction of both furca and retinaculum.

#### Description.

*Holotype* length 0.67 mm. Intravital colour unknown, holotype stored in alcohol for more than 30 years completely lacks dark pigment even on the eye fields. Tegument granulations rather fine and uniform. Ant.4 with a simple apical vesicle and four blunt sensilla (one dorsal and three lateral), rather short and subequal in size, both subapical microsensillum and organite invisible due to the poor condition of the slide. AO typical of the genus, outer sensilla rather short. Ant.1 and Ant.2 with seven and eleven setae, respectively.

*Head* with 5+5 subequal ocelli, as usual. Buccal cone typical of the genus, not elongate. Setal formula of labrum, 4/554, setae of distal row clearly thickened. Labium with all common papillae (A–E), 9 guards (five long and four short, truncate and papillate) and six proximal setae. Basomedial field of labium (submentum) with four setae, basolateral field (mentum) with five setae, as usual. Maxillary outer lobe simple, with one sublobal seta.

**Figures 30–35.**
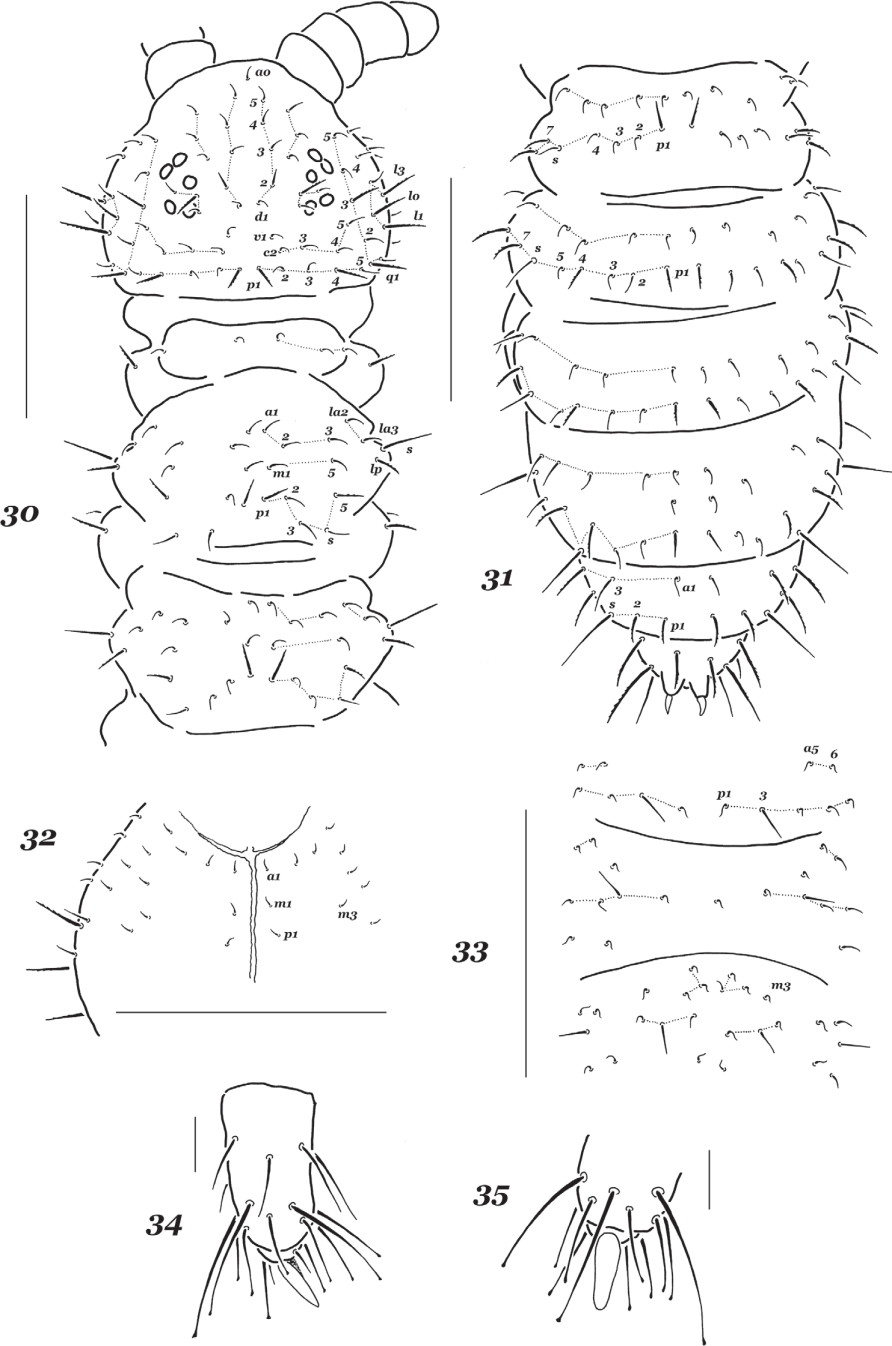
*Xenylla aculeata* sp. nov. **30** dorsal chaetotaxy of head and thorax **31** dorsal chaetotaxy of abdomen **32** ventral chaetotaxy of head **33** ventral chaetotaxy of Abd.2–4 **34, 35** tip of tibiotarsus, different views. Scale bars: 0.1 mm (**30–33**), 0.01 mm (**34, 35**).

*Most dorsal setae* stout, finely ciliate and clearly differentiated into finer microsetae and subspiniform macrosetae, sensilla usually long and straight, especially on abdominal tip and laterally on Th.2 and Th.3 (Figs 30–31). Head with all usual dorsal setae present, except for c1 [b]; setae p1, p4, d2, oc2, q1, q3, l0, l1, and l3 more or less clear macrosetae, l1 only slightly longer than l3 (ratio = 1.1–1.2/1). Th.2 and Th.3 with seta a2 in a posterior position compared to a1 [h1], both p1 and p2 setae set in front of p3, setae la1 and m3 absent [i and k]; setae p1, p5 and p6 being macrosetae, lateral sensilla long, but dorsal ones rather short (Fig. 30). Chaetotaxy of abdominal terga as follows: Abd.1 without p5, m3 setae absent from Abd.4 [o] and a2 from Abd.5 [q] (Fig. 31). Ventral chaetotaxy: head with setae p1 and m3 (Fig. 32), Th.2 and Th. 3 with 1+1 axial setae, abdominal sterna as in Fig. 34, Abd.2 with p1, but without p2, Abd.3 with a medial unpaired seta, Abd.4 with only one m-seta (m1 and m2 absent [a4 and a5]). General code of chaetom: bh_1_ikoqa_4_a_5_.

*Ventral tube* with 4+4 setae. furca and retinaculum completely absent. Tibiotarsi of legs 1–3 with 19, 19, and 18 setae, respectively; all setae of distal whorls (A+T) more or less clearly clavate (Figs 34, 35). Anal spines rather long and set on subequal cuticular papillae (Fig. 31).

#### Etymology.

The name of the new species is derived from the Latin *aculeata*, meaning spiny, to reflect the shape of the dorsal macrosetae characteristic of *X. aculeata* sp. nov.

#### Affinities.

The chaetotaxic pattern of *X. aculeata* sp. nov. allows us to include it into group VI (b), although a forward position of both p1 and p2 on Th. 2 and Th.3 appears to be unique to the new species. There is only one other species in the group that has neither a furca nor a retinaculum, viz. *X. osetica* Stebaeva & Potapov, 1994. Yet the latter species can easily be distinguished from *X. aculeata* sp. nov. by the presence of three sublobal setae on the maxillary outer lobe (vs. one in *X. aculeata* sp. nov.) and a complete chaetotaxy of Th.2 and Th.3 with setae al1 and m3 being present (absent in *X. aculeata* sp. nov.). Thus latter character (the absence of setae) seems to point to the relations between *X. aculeata* sp. nov. and several other congeners of the same group that share a functional furca with one (*X. betulae* Fjellberg, 1985) or two dental setae (*X. corticalis* Börner, 1901, *X. grisea* Axelson, 1900, *X. hexagona* Fjellberg, 1992, and *X. laurisilvae* Fjellberg, 1992). Among these species, only *X. betulae* has hitherto been known from the region under study. It appears to be rather similar to the new species in having an identical dorsal chaetotaxy including such fine characters as short sensilla in p-row on Th.2–Abd.1 and the usual absence of setae p5 from Abd.1 and of setae p7 from Abd.4. Ventral chaetotaxy (the absence of p2 from Abd.2 and of some m-setae from Abd.4, the presence of axial unpaired setae on Abd.3), the rather strong anal spines, and the long stout setae on the abdominal tip are also similar. Apart from the complete absence of a furca, *X. aculeata* sp. nov. differs from *X. betulae* in having much finer tegument granulation, coarser and more clearly differentiated dorsal setae, only one sublobal seta on the maxillary outer lobe (vs. two sublobals in *X. betulae*), p1-setae in an anterior position on Th.2 and Th.3, and numerous clavate setae on tibiotarsi (vs. 2–2–2 in *X. betulae*). The latter character appears to be unique in the genus, but needs verification based on fresh material.

#### Distribution and ecology.

The only known specimen of *X. aculeata* sp. nov. was found in a typical swampy association of the region, but a search in similar communities in the same area failed to reveal additional material. Taking this into account, as well as some morphological traits of the new species, namely the complete reduction of a furca and the presence of numerous clavate tibiotarsal setae, *X. aculeata* sp. nov. can be assumed to rather represent a corticicolous, not hygrophilous, species.

### Xenylla arnei sp. nov.

Taxon classificationAnimaliaPoduromorphaHypogastruridae

23C640A4-C3D1-538A-9712-87779004E869

http://zoobank.org/4BB2F510-C7FE-4BDD-9412-7076B364C4D8

Figs 36–45 , 46–47

#### Type material.

***Holotype*** Russia, North-East • ♂; Magadan Province, Tauisk; 59°43.66'N, 149°21.85'E; coastal meadow; July 2017; M. Bizin and B. Efeykin leg.

***Paratypes*** Russia, North-East • 5 ♀♀ and 5 ♂♂, same data as for holotype. The types are deposited in MSPU.

#### Additional material.

more than 500 specimens (alcohol), mainly from the holotype locality; Several specimens from this material were sequenced (Table 1). Their partial COI genes were amplified and deposited in the GenBank under the sample ID: KY066787–KY066789; 9 specimens; same region, AF-51/79: “Geartner Bay, steep slope down to beach with *Artemisia*, *Sedum*, *Saxifraga*, *Potentilla*, grasses, 20.07.1979”; A. Fjellberg leg.

#### Diagnosis.

A species from chaetotaxic group II of the genus *Xenylla* with a general chaetotaxic code of h_1_rt, characterised by a light brownish colour, non-differentiated dorsal setae, and the absence of a prominent cuticular lobe from the subcoxae of hind legs.

#### Description.

Length 1.4–1.7 mm. Colour rather light, yellow-brown (chamois), with patches of diffuse darker pigmentation, ocular field and antennal tip dark, ventral side usually paler. Tegument granulation fine and almost uniform. Ant.4 with a simple apical vesicle and four blunt sensilla (one dorsal [S3 or S4?] and three lateral [S7–S9]), relatively short and subequal in size, both a subapical microsensillum and an organite are present. AO typical of the genus, outer sensilla thinner than subapical ones, but not especially short (1:1.2–1.8, Fig. 39). Ant.1 and Ant.2 with seven and 12 or 13 setae, respectively.

*Head* with 5+5 subequal ocelli, as usual. Buccal cone typical of the genus, not elongate. Setal formula of labrum, 4/554, setae of distal row clearly thickened. Labium with all common papillae (A–E), 12 guards (eight long and four short, rhabdoid, papillate) and six proximal setae (Fig. 38). Basomedial field of labium (submentum) with four setae, basolateral field (mentum) with five setae, as usual. Maxillary outer lobe simple, with three sublobal setae.

**Figures 36–45.**
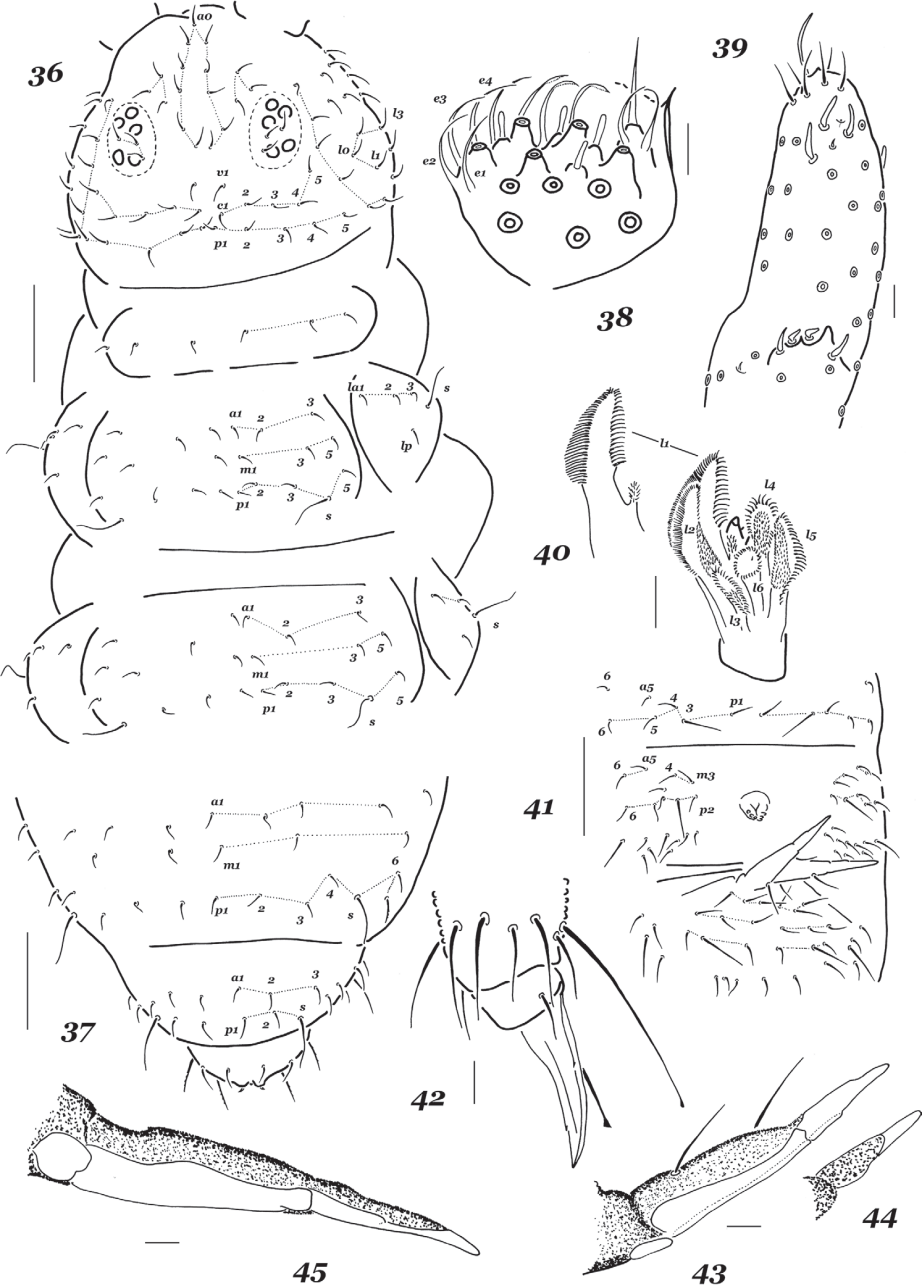
*Xenylla arnei* sp. nov. **36** dorsal chaetotaxy of head and thorax **37** dorsal chaetotaxy of Abd.4–6 **38** labial palp **39** sensorial equipment of Ant.3 and Ant.4 **40** maxillary head **41** ventral chaetotaxy of Abd.2 and Abd.3 **42** tip of leg 3 **43** dens and mucro, lateral view **44** mucro, dorsal view **45** dens and mucro, ventral view. Scale bars: 0.1 mm (**36, 37, 41**), 0.01 mm (**38–40, 42–45**).

**Figures 46–50.**
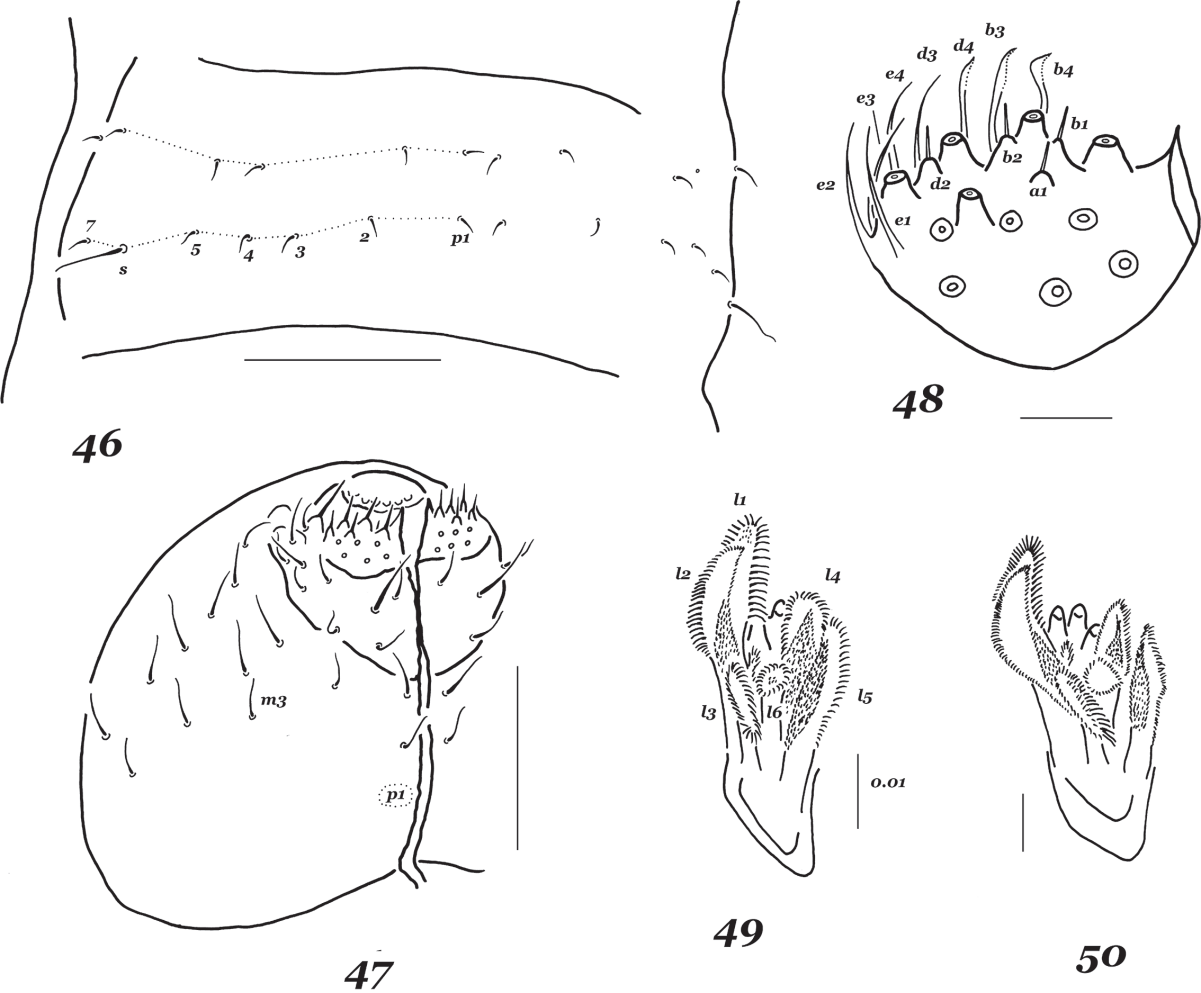
*Xenylla arnei* sp. nov. (**46, 47**) and *X. humicola* (**48–50**) **46** dorsal chaetotaxy of Abd.1 **47** ventral chaetotaxy of head **48** labial palp **49** maxillary head, specimen from Kola Peninsula **50** maxillary head, specimen from Chukotka. Scale bars: 0.1 mm (**46, 47**), 0.01 mm (**48–50**)

*Dorsal setae* fine, thin, and barely differentiated (except for those on Abd.6), tergal sensilla clearly longer than ordinary setae (~ 3.2–3.4:1), dorsal and lateral sensilla on Th.2 and Th.3 subequal. Head with a basic set of setae (Fig. 37), lateral setae not differentiated. Th.2 and Th.3 also with all usual setae, a2 in a posterior position compared to a1 [h1] only on Th.3, p2 seta set aligned with p1 and p3 (Fig. 36). Abdominal terga also with basic set of setae (Fig. 37), p-row on Abd.1–3 with p5 present, s = p6 (Fig. 46). Ventral chaetotaxy: head with 2+2 setae along midline (p1 absent [r]), m3 present (Fig. 48), Th.2 and Th.3 without axial setae [t]. Abdominal sterna as in Fig. 41: Abd.2 with p1 but without p2, Abd.3 without medial unpaired seta above retinaculum. General chaetotaxic code as h_1_rt.

*Ventral tube* with 4+4 setae. Retinaculum with 3+3 teeth. Furca complete, both dens and mucro thin, long, and clearly separated ventrally. Mucrodens/U_3_ ratio as 2.6–2.9:1. Dens with two dorsal setae (Fig. 43), ventral side of dens and mucro completely smooth, without primary granulations (Figs 44, 45). Mucro shorter than dens (0.7–0.8:1), with a low outer lamella, ventral thickening neither prominent nor with a clear tooth. Chaetotaxy of legs 1–3 usually as follows: upper subcoxae – 1, 3, 3; lower subcoxae – 0, 3, 3; coxae – 3, 8, 8; trochanters – 6, 6, 6; femora – 13, 12, 11; tibiotarsi – 19, 19, 18 setae, respectively. Tibiotarsal setae A1 and A7 on all legs as long as 1.5–1.7 inner edge of unguis, clearly clavate. Unguis with a pair of lateral teeth and usually with a small tooth in upper third of inner edge. Anal spines short, usually curved and set on tiny cuticular papillae.

#### Etymology.

The new species is named after the famous Norwegian collembologist, Arne Fjellberg, who discovered it in the Magadan Region more than 40 years ago.

#### Affinities.

Using the most recent key to the Asiatic species of the genus (Jia and Skarżyński 2019), *X. arnei* sp. nov. keys out to *X. humicola* (Fabricius, 1790), because their general chaetotaxic codes [h_1_rt] are identical. Moreover, both species are similar in many other important morphological traits, namely the labial palp is with four e-guards (cf. Fig. 38 and Fig. 48), which is not typical of the genus [see Fjellberg (1999)], the virtually identical maxillae (cf. Fig. 40 and Figs 49, 50), three sublobals on the maxillary outer lobe, a retinaculum with 3+3 teeth, a long dens with two setae and a narrow straight mucro. Nevertheless, several fine morphological differences between *X. arnei* sp. nov. and *X. humicola* are traceable. Apart from the different colouration, the non-differentiated setae l1 and l3 on the head (vs. similar in size, but spine-like setae in *X. humicola*) and the absence of subcoxal lobes from Th.3 in *X. arnei* sp. nov. (variable in shape, but always present in *X. humicola*) seem to be the most sound. In addition, the species status of *X. arnei* sp. nov. is well confirmed by molecular evidence (Table 4).

**Table 4.** K2P distances in *Xenylla* species from GenBank and our sequences, measured in %.

	Species	Region	1	2	3	4	5	6	7	8	9	10	11	12	13
1	*X. humicola*	Magadan													
2	*X. humicola*	Vaigach	0.6												
3	*X. humicola*	Estonia	0.9	1.2											
4	*X. betulae*	Ontario	28.0	27.9	30.1										
5	*X. boerneri*	UK	25.0	25.4	23.1	28.6									
6	*X. brevisimilis*	Ontario	27.5	27.1	26.4	24.8	25.8								
7	*X. canadensis*	Ontario	30.4	30.8	28.8	26.2	31.2	30.7							
8	*X. grisea*	Antarctica	25.7	25.4	24.0	32.3	23.7	26.1	26.7						
9	*X. maritima*	Ontario	22.3	22.7	21.1	34.9	18.8	30.3	34.5	27.4					
10	*X. mediterranea*	Ontario	28.1	28.5	26.2	27.9	30.4	29.1	27.2	23.9	26.3				
11	*X. pomorskii*	Poland	28.6	28.2	29.5	24.6	25.7	24.7	28.7	28.7	29.6	27.1			
12	*X. szeptyckii*	Poland	22.7	23.1	24.6	30.0	23.7	28.0	33.5	24.7	22.2	28.9	28.7		
13	*X. arnei* sp. nov.	Magadan	22.9	22.6	23.4	29.1	22.2	22.9	25.9	25.2	26.1	26.7	24.4	25.3	
14	*X. tullbergi*	France	22.4	22.8	23.1	28.7	24.5	27.7	31.0	24.7	22.1	27.8	28.3	19.1	22.7

*Xenylla arnei* sp. nov., together with the widespread *X. humicola* and the Japanese *X. brevispina* Kinoshita, 1916 [h_1_rtsv], represent the only known Holarctic members of the species group II (Stebaeva and Potapov 1994). There is also one more form, *X. convexopyga* Lee, Park & Park, 2005, from the Korean Peninsula, in which the chaetotaxy is rather similar. The chaetotaxic code given for this species in the original description is as follows: krtsv. This code is likely to be not fully correct. The absence of a m3 seta from Th.2 [k] contradicts to fig. 1A in Lee et al. (2005), where this seta is present, albeit marked as m4 (a situation unknown within the genus). In our view, the species rank of *X. convexopyga* and its separation from *X. brevispina* need confirmation.

All other species of this group, *viz. X. yukatana* Mills, 1938 [h_1_tiqa3a4], *X. gamae* Cardoso, 1968 [h_1_tiq], *X. nigeriana* Gama & Lasebikan, 1976 [h_1_tiqsoa_3_], *X. brasiliensis* Gama, 1978 [h_1_rtlq], and *X. nirae* Gama & Oliveira, 1994 [h_1_rtlqiomu], are characterised by more strongly reduced chaetotaxy patterns and inhabit various tropical regions.

#### Molecular data.

GenBank currently contains COI sequences for only eleven species of the genus (of 140 species known worldwide). The obtained interspecific divergences (Table 4) between all of them, together with *X. arnei* sp. nov., range between 18.8 to 34.9% (mean 26.5%). Analogous data for *X. arnei* sp. nov. are 22.2–29.1% (mean 24.9%), which can be considered as an additional argument in favour of its specific status. Nevertheless, such a scant amount of primary data does not allow for any serious statements to be made, but a trend to the absence of parallelisms in molecular and morphological evolution can be traced quite clearly.

#### Distribution and ecology.

*Xenylla arnei* sp. nov. was collected in two neighbouring sites located on the northern shore of the Sea of Okhotsk, in the vicinity of Magadan. In this region, it was mainly found in various herbaceous meadows at some distance off the coastal line, where its abundance may be very high. Its occurrence in all other types of coastal plat associations in the study area was rather sporadic.

## Supplementary Material

XML Treatment for Hypogastrura variata

XML Treatment for Hypogastrura yosii

XML Treatment for Xenylla aculeata

XML Treatment for Xenylla arnei
