# Corrigenda: Three new and one little-known species of Hypogastruridae (Collembola) from Russia’s northeast. ZooKeys 1005: 1–20. https://doi.org/10.3897/zookeys.1005.54882

**DOI:** 10.3897/zookeys.1028.65169

**Published:** 2021-04-06

**Authors:** Anatoly Babenko, Boris Efeykin, Mikhail Bizin

**Affiliations:** 1 Severtsov Institute of Ecology & Evolution, Russian Academy of Sciences, Leninski pr. 33, 119071, Moscow, Russia Severtsov Institute of Ecology & Evolution, Russian Academy of Sciences Moscow Russia; 2 Kharkevich Institute for Information Transmission Problems, Russian Academy of Sciences, Bol’shoi Karetnyi per. 19, 127051, Moscow, Russia Kharkevich Institute for Information Transmission Problems, Russian Academy of Sciences Moscow Russia

## Abstract

none

After our recent publication (Babenko et al. 2020), Dr. Cyrille A. D’Haese, of the Muséum national d’Histoire naturelle, Paris, France, called our attention to the errors contained in Table 1 (column: COI sequence number). We would like to take this opportunity to sincerely thank him and correct the following errors:

**Table 1.** Species used for molecular study, primers, and GenBank accession numbers of the sequences.

Species	Forward primer	Reverse primer	COI sequence number	Sequence size
*Hypogastrura variata* sp. nov.	colfol-for: tttcaacaaatcataargayatygg	colfol-rev: taaacttcnggrtgnccaaaaaatca	**MW518020**	647 bp
			**MW518021**	
			**MW518022**	
*Hypogastrura yosii* Stach, 1964	colfol-for: tttcaacaaatcataargayatygg	colfol-rev: taaacttcnggrtgnccaaaaaatca	**MW507473**	647 bp
			**MW507474**	
			**MW507475**	
*Xenylla arnei* sp. nov.	LCO1490_t1: tgtaaaacgacggccagtgg tcaacaaatcataaagatattgg	HCO2198_t1: caggaaacagctatgactaaacttc agggtgaccaaaaaatca	**MW517749**	658 bp
			**MW549335**	
			**MW549336**	

Other corrections:

**Page 3, Additional material** to *Hypogastrura variata* sp. nov., lines 3–4: Their partial COI genes were… deposited in the GenBank under the sample ID: MW518020– MW518022.

**Page 8, Material** to *Hypogastrura yosii* Stach, 1964, lines 3–4: Their partial COI genes were… deposited in the GenBank under the sample ID: MW507473– MW507475.

**Page 13, Additional material** to *Xenylla arnei* sp. nov., lines 2–4: Their partial COI genes were…deposited in the GenBank under the sample ID: **MW517749, MW549335**–MW549336.
